# The Impact of Dietary Fat on Breast Cancer Incidence and Survival: A Systematic Review

**DOI:** 10.7759/cureus.30003

**Published:** 2022-10-06

**Authors:** Ankit Gopinath, Ameer Haider Cheema, Keyur Chaludiya, Maham Khalid, Marcellina Nwosu, Walter Y Agyeman, Aakash Bisht, Sathish Venugopal

**Affiliations:** 1 Internal Medicine, California Institute of Behavioral Neurosciences & Psychology, Fairfield, USA; 2 Neurology, California Institute of Behavioral Neurosciences & Psychology, Fairfield, USA

**Keywords:** breast cancer prognosis, causes of breast cancer, breast cancer mortality, fat, breast cancer survival, breast cancer risk, saturated fat, dietary fat, breast cancer

## Abstract

Breast cancer has become one of the most common cancers affecting women worldwide, and researchers have struggled to explore new avenues for managing it for decades. One of the ways was to analyze the diet and its importance concerning this disease. This review aims to study the effect dietary fat has on the risk of breast cancer incidence as well as its influence on the survival of breast cancer patients. The main population under consideration for this review is women older than the age of 18.

A thorough and detailed search was conducted until July 21, 2022, using four databases: PubMed, ScienceDirect, Google Scholar, and The Cochrane Library. After screening, 22 articles were selected for inclusion in this review. Of the 22 articles, 12 were from PubMed, two from ScienceDirect, one from The Cochrane Library, and seven from Google Scholar. The risk of bias was assessed, and the required information was extracted from the articles.

A systematic review of all the included articles found a significant correlation between dietary fat and an increased risk of breast cancer development and worsening the prognosis for patients already diagnosed with breast cancer.

Although many overlapping factors may be responsible for this development, studies show a trend that suggests that this particular factor can be a contributor. Further studies need to be conducted to highlight the role of fat in the diet and the use of dietary modification to curb breast cancer rates.

## Introduction and background

In 2020, according to statistics by GLOBOCAN (Global Cancer Observatory), there were over two million cases of breast cancer diagnosed worldwide, which comprised 25% of all cancers among women [1]. More recently, it is projected that in 2022, breast cancer will account for 30% of those numbers among newly diagnosed cancers in females. The World Health Organization (WHO) stated that breast cancer had become one of the most prevalent cancer subtypes globally as of 2021, comprising 12% of all newly diagnosed cases annually [2]. The mortality rates of breast cancer have become so significant that it has become one of the top five leading causes of cancer mortality in women across the globe [3]. This has resulted in over 685,000 deaths [1].

Multiple modifiable risk factors for breast cancer have been recognized, such as exposure to radiation, alcohol consumption, obesity, hormonal causes, and insufficient physical activity [3]. Among these causes, one of the most impactful factors identified is lifestyle [4].

Evolving evidence suggests that diet, one of the most important components of a healthy lifestyle, plays a crucial part in its development [3]. As another risk factor that can be modified and which contributes to approximately 35% of all cancer cases, it has become increasingly vital to map out and control the role of diet in cancer pathogenesis [5].

In many previously conducted studies regarding causative factors for diet-induced breast cancer, both nutrition, dietary patterns, and individual foods have been considered. For example, elevated levels of consumption of processed meats, alcohol, and animal fats, and decreased levels of dietary fiber, fruits, and vegetables may be associated with a greater risk of developing breast cancer [5].

Among important nutrient groups, fat has garnered increasing attention for having some association with breast cancer [1]. Biochemically, fats can be described as molecules with a glycerol backbone and three independent fatty acid tails connected by an ester linkage [6]. Many case-control studies conducted in the past have shown a close association between total fat intake in the diet and breast cancer [1]. A meta-analysis has even shown that women with a higher total fat intake had as much as a 13% increased risk of developing breast cancer than those who consumed relatively less [7].

This systematic review will pursue the correlation between dietary fat and breast cancer, highlighting the possible causation and effect that a diet that is rich in fat has on the incidence of breast cancer and potential implications for mortality among breast cancer patients.

## Review

Methods

A systematic literature search was conducted under the Preferred Reporting Items for Systematic Reviews and Meta-Analysis (PRISMA) guidelines [8].

A free full-text article search was instituted utilizing databases such as PubMed, Google Scholar, ScienceDirect, and the Cochrane Library between the dates of June 5, 2022, and July 21, 2022. The keywords "dietary fat," "breast cancer," and "mortality" were used in addition to Medical Subject Headings (MeSH) terms, either alone or in combination.

Inclusion and Exclusion Criteria

Relevant articles were selected based on keywords and MeSH search terms. Only free full-text articles in English that were published within the last 20 years and that included women beyond the age of 18 have been selected. Articles were excluded from non-English publications and studies involving males and animals.

PubMed Search Strategy

The detailed MeSH search strategy in PubMed is given in Table 1.

**Table 1:** PubMed search strategy MeSH (Medical Subject Headings)

MeSH term	Results without filtering for eligibility criteria	Results filtering for eligibility criteria
Dietary Fat OR (( "Dietary Fats/administration and dosage"[Mesh] OR "Dietary Fats/adverse effects"[Mesh] OR "Dietary Fats/analysis"[Mesh] OR "Dietary Fats/genetics"[Mesh] OR "Dietary Fats/immunology"[Mesh] OR "Dietary Fats/metabolism"[Mesh] OR "Dietary Fats/statistics and numerical data"[Mesh] OR "Dietary Fats/supply and distribution"[Mesh] OR "Dietary Fats/therapy"[Mesh] )) AND ( "Dietary Fats/administration and dosage"[Majr:NoExp] OR "Dietary Fats/adverse effects"[Majr:NoExp] OR "Dietary Fats/analysis"[Majr:NoExp] OR "Dietary Fats/genetics"[Majr:NoExp] OR "Dietary Fats/immunology"[Majr:NoExp] OR "Dietary Fats/metabolism"[Majr:NoExp] OR "Dietary Fats/statistics and numerical data"[Majr:NoExp] OR "Dietary Fats/supply and distribution"[Majr:NoExp] OR "Dietary Fats/therapy"[Majr:NoExp] )	15,741	176
Breast Cancer OR breast neoplasm OR ( "Breast Neoplasms/analysis"[Majr:NoExp] OR "Breast Neoplasms/chemically induced"[Majr:NoExp] OR "Breast Neoplasms/diet therapy"[Majr:NoExp] OR "Breast Neoplasms/etiology"[Majr:NoExp] OR "Breast Neoplasms/genetics"[Majr:NoExp] OR "Breast Neoplasms/immunology"[Majr:NoExp] OR "Breast Neoplasms/metabolism"[Majr:NoExp] OR "Breast Neoplasms/mortality"[Majr:NoExp] OR "Breast Neoplasms/statistics and numerical data"[Majr:NoExp] )	468,148	1,624
Mortality OR ( "Mortality/drug effects"[Mesh] OR "Mortality/epidemiology"[Mesh] OR "Mortality/ethnology"[Mesh] OR "Mortality/etiology"[Mesh] OR "Mortality/prevention and control"[Mesh] OR "Mortality/statistics and numerical data"[Mesh] OR "Mortality/trends"[Mesh] )	1,450,515	6,017

Combined search results after screening by title and abstract on PubMed: 12 results.

Date of the last search: July 21, 2022.

Search Strategies in Other Databases

The databases of Google Scholar, ScienceDirect, and The Cochrane Library were searched using the keywords "dietary fat," "breast cancer," and "mortality." The results are shown in Table 2.

**Table 2:** Combined database search results for Google Scholar, ScienceDirect, and The Cochrane Library

Database	Results without filtering for eligibility criteria	Results filtering for eligibility criteria	Results after screening for relevance
Google Scholar	4872	369	7
The Cochrane Library	34	11	1
ScienceDirect	319	17	7

Date of the last search: July 21, 2022.

Results

A total of 1282 articles were found within the eligibility criteria by utilizing keywords and MeSH terms. One duplicate was found in the PubMed database, and 1281 articles were then screened for relevance.

Of the 1281 articles, 584 were from PubMed, 369 were from Google Scholar, 11 were from the Cochrane Library, and 317 were from ScienceDirect. The screening was then conducted by title and abstract, as well as relevance to the review. This involved screening specifically for articles that show a correlation between dietary fat and breast cancer. The final selection consisted of 22 articles, which were included in this review.

**Figure 1:**
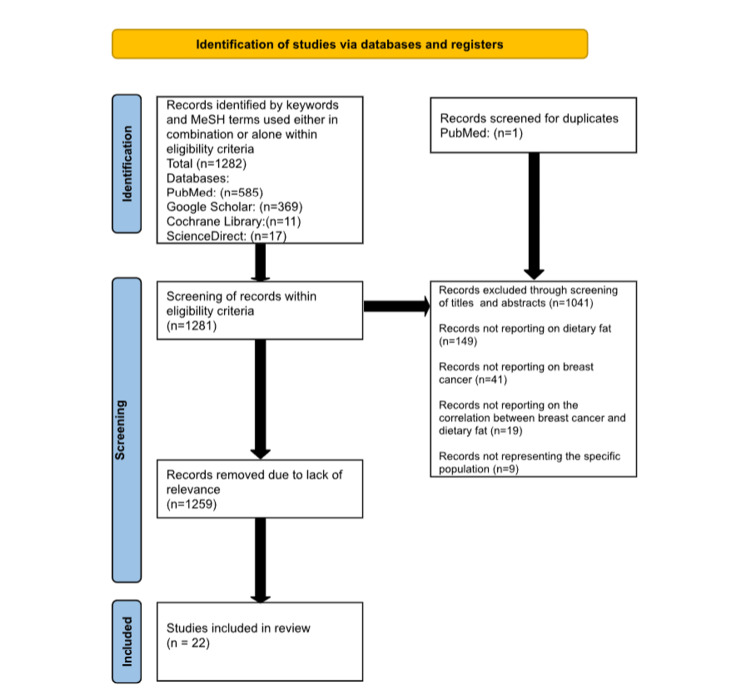
PRISMA flowchart of the search strategy

The PRISMA flowchart of the literature and search strategy is shown in Figure 1 [8].

Study Characteristics

The Scale for the Assessment of Narrative Review Articles (SANRA) [9] was used for the quality appraisal of narrative literature reviews. For this tool, the minimum threshold for good-quality articles was kept at eight. The maximum possible score is 12. The results, along with the study characteristics, are shown in Table 3.

**Table 3:** Study characteristics of reviews and their SANRA score SANRA: Scale for the Assessment of Narrative Review Articles

Author/ Year of publication	Study	Type of study	Interpretation	SANRA score
Blucher et al. [10] (2017)	Obesity and Breast Cancer: Current Insights on the Role of Fatty Acids and Lipid Metabolism in Promoting Breast Cancer Growth and Progression	Review	Extracellular lipids promote the growth and progression of breast cancer.	9/12, Good
Tan et al. [11] (2019)	Effect of High-Fat Diets on Oxidative Stress, Cellular Inflammatory Response and Cognitive Function	Review	High-fat diets can induce oxidative stress.	10/12, Good
Sellami et al. [12] (2020)	Nutrigenomics and Breast Cancer: State-of-Art, Future Perspectives and Insights for Prevention	Review	There is a correlation between breast cancer and a non-alcohol-related diet.	10/12, Good
Shapira [13] (2017)	The potential contribution of dietary factors to breast cancer prevention	Review	Lifelong dietary intervention can help reduce breast cancer incidence.	10/12, Good
De Cicco et al. [14] (2019)	Nutrition and Breast Cancer: A Literature Review on Prevention, Treatment and Recurrence	Review	A healthy dietary pattern can improve breast cancer prognosis and quality of life.	10/12, Good
Garcia-Estevez et al. [4] (2019)	Updating the role of obesity and cholesterol in breast cancer	Review	Obesity, dietary fat, and cholesterol affect the onset and progression of breast cancer.	10/12, Good
Cenker [15] (2019)	Dietary Fat Consumption And Breast Cancer: A Review Of The Literature	Review	Low-fat or Mediterranean dietary patterns reduce the risk of breast cancer.	10/12, Good

The Assessment of Multiple Systematic Reviews (AMSTAR) [16] checklist was used to assess systematic reviews with meta-analyses, with 12 being the minimum score needed out of a maximum possible score of 16. The results and study characteristics are shown in Table 4.

**Table 4:** Study characteristics and AMSTAR quality appraisal for systematic reviews with meta-analysis RR - Relative risk CI - Confidence interval p - P-factor

Author/Year of publication	Study	Number of studies included in the meta-analysis	Study population size (Women)	Conclusion	Results	AMSTAR score
Lodi et al. [17] (2022)	Lipid Intake and Breast Cancer Risk: Is There a Link? A New Focus and Meta-Analysis	44 studies (28 case-control and 16 cohort studies)	1,185,896	A weak association between breast cancer risk in postmenopausal women and a high saturated fat diet was found.	Case-control studies (RR = 1.12, 95% CI = 1.03–1.21, p = 0.006); cohort studies (RR = 1.01, 95% CI = 0.85–1.19, p = 0.93).	13/16, Good
Yongjo kim et al. [6] (2020)	Association between dietary fat intake and mortality from all causes, cardiovascular disease, and cancer: A systematic review and meta-analysis of prospective cohort studies	19 cohort studies	1,013,273	Diets high in saturated fat had a high all-cause mortality rate, including cancer.	Every 5% increase in saturated fat intake increases the risk of cancer mortality by 4% (RR = 0.93; 95% CI = 0.89-0.97).	14/16, Good
Brennan et al. [18] (2017)	Dietary fat and breast cancer mortality: A systematic review and meta-analysis	15 prospective cohort studies	29241	Saturated fat intake has a negative association with the survival of breast cancer patients.	Women in the highest vs. lowest category of intake of saturated fat had a higher rate of breast cancer-specific death (HR = 1.51, 95% CI = 1.09-2.09, p 0.01).	14/16, Good
Shetty et al. [7] (2019)	Breast Cancer and Dietary Fat Intake: A correlational study	88 cross-sectional studies	Not available	Increased total fat consumption increases the risk of breast cancer.	A statistically significant correlation (p<0.001) of more than 0.6 between fat consumption and crude breast cancer incidence rates was found.	12/16, Good
Smith-Warner et al. [19] (2001)	Types of dietary fat and breast cancer: a pooled analysis of cohort studies	Eight prospective cohort studies	351,821	Weak positive association for substituting saturated fat for carbohydrates in breast cancer risk.	(RR = 1.09 for an increment of 5% of energy from saturated fat, 95% CI = 1.00–1.19)	12/16, Good
Boyd et al. [20] (2003)	Dietary fat and breast cancer risk revisited: a meta-analysis of the published literature	31 case-control and 14 cohort studies	603,795	In case-control and cohort studies, total fat and saturated fat were significantly associated with breast cancer risk.	Total fat: RR=1.13 (95% CI= 1.03–1.25) Saturated fat: RR = 1.19 (95% CI = 1.06–1.35).	13/16, Good
Xia et al. [21] (2015)	Meta-Analysis of Saturated Fatty Acid Intake and Breast Cancer Risk	28 case-control and 24 cohort studies	~50,000	Dietary saturated fat is linked with breast cancer risk.	Case-control studies (high vs. low intake): RR = 1.18 (95% CI = 1.03-1.34); cohort studies (high vs. low intake, RR = 1.04 (95% CI = 0.97-1.11).	13/16, Good

The characteristics and appraisal of the clinical trials selected for discussion in this review are shown in Table 5.

**Table 5:** The characteristics and appraisal of the clinical trial HR - Hazard ratio) CI - Confidence interval

Author/Year of publication	Study	Clinical trial type	Number of participants (women)	Age demographic	Result	Interpretation
Chlebowski et al. [22] (2019)	Low-fat dietary pattern and long-term breast cancer incidence and mortality: The Women’s Health Initiative randomized clinical trial	randomized, controlled	48,835	50-79 years	HR= 0.79,95%, CI= 0.64-0.97	A significant decrease in the risk of death from breast cancer with a low-fat dietary pattern
Murillo-Ortiz et al. [23] (2017)	Effect of reduced dietary fat on estradiol, adiponectin, and IGF-1 levels in postmenopausal women with breast cancer	randomized, controlled	100	The mean age of Group A is 50.45 (7.94) years, and the mean age of Group B is 52.26 (6.11) years.	Control diet vs. low fat diet group: Estradiol levels= 21.23 (± 14.32} vs. 16.05 (± 10.25) ng/mL; p < 0.001)	A low-fat diet shows a decrease in levels of estradiol, lowering breast cancer risk.

The Cochrane risk-of-bias assessment tool was used to assess the quality of the clinical trials, as shown in Table 6.

**Table 6:** The Cochrane risk-of-bias assessment tool

Clinical trial	Selection bias	Reporting bias	Performance bias	Detection bias	Attrition bias
Chlebowski et al. [22]	Low risk	Low risk	Low risk	Low risk	Low risk
Murillo-Ortiz et al. [23]	Low risk	Low risk	Low risk	Low risk	Low risk

Table 7 shows the data collected from observational studies that were selected for this review.

**Table 7:** The characteristics of observational studies

Author/Year of publication	Study	Study type	Population size (Women)	Age demographic	Result
Stasiewicz et al. [1] / 2022	Dietary Fat Intake: Associations with Dietary Patterns and Postmenopausal Breast Cancer—A Case-Control Study	Case-control	420	50-79 years	An increase in dietary fat (greater than 32% energy intake) was associated with increased breast cancer in perimenopausal and postmenopausal women.
Kim et al. [5] / 2017	Dietary Factors and Female Breast Cancer Risk: A Prospective Cohort Study	Prospective cohort	5046	>30 years	The consumption of grilled meat and a high-cholesterol diet are associated with a higher risk of breast cancer.
Mialich et al. [24] / 2018	Assessment of the nutritional and metabolic profile of women with breast cancer and its association with metabolic syndrome	Prospective cohort	224	32-85 years	High body fat and body mass index (BMI) have a strong association with breast cancer patients.
Shahril et al. [25] / 2021	‘Energy-Dense, High-SFA and Low-Fiber’ Dietary Pattern Lowered Adiponectin but Not Leptin Concentration of Breast Cancer Survivors	Cross-sectional	128	32-72 years	Linkage of a high saturated fat diet with low adiponectin and excess body weight in breast cancer patients.
Sieri et al.) [26] / 2014	Dietary Fat Intake and Development of Specific Breast Cancer Subtypes	Prospective cohort	337327	>18 years	Elevated total and saturated fats are associated with a greater risk of estrogen receptor and progesterone receptor-positive breast cancer.
Sofi et al. [27] / 2018	Nutritional risk factors and status of serum 25(OH)D levels in patients with breast cancer: A case control study in India	Case-control	200	Cases = 45(±9) years, Controls = 46(±10) years	Saturated fat consumption was linked to an increased risk of breast cancer.

The Newcastle-Ottawa Scale was used to assess the quality of observational studies. The minimum acceptable score was six out of a possible eight to be included in this review. The results are shown in Table 8.

**Table 8:** Newcastle-Ottawa Scale assessment of observational studies

Study	Selection	Comparability	Exposure/Outcome	Overall score
Stasiewicz et al. [1]	4	1	3	8, Good
Kim et al. [5]	3	1	3	7, Good
Mialich et al. [24]	3	1	3	6, Good
Shahril et al. [25]	3	1	2	6, Good
Sieri et al.) [26]	3	1	3	7, Good
Sofi et al. [27]	3	1	2	6, Good

Discussion

To understand the association between dietary fat and breast cancer (BC), let us first look at the pathophysiology of fat intake in our diet and the causation of breast cancer.

Multiple hypothesized pathways are associated with fat consumption and cancer onset. One such pathway is the mechanism by which they are both linked: oxidative stress and the production of reactive oxygen species (ROS). ROS are free radicals that are highly reactive and interact with the DNA within our body’s cells. ROS can cause DNA damage and changes in gene expression, potentially leading to cancer-causing genes such as oncogenes.There could then be a change in cell signaling pathways, causing a cascade effect and prompting carcinogenesis [14] by changing the rate of cell growth, proliferation, and cell death [1]. A high-fat diet can cause an increase in chylomicrons in the intestine, causing an increase in free fatty acids (FFA). FFA can then be sent to the mitochondria for β-oxidation. The cytochrome c oxidase component of the electron transport chain then increases the electron flow, causing ROS accumulation [11].

Fat consumption may also have an indirect connection with breast cancer through another major disease epidemic in the world, obesity. Obesity refers to a condition where individuals have a high amount of adipose tissue. Numerous studies have shown a positive correlation between adiposity and a high-fat diet (relative risk (RR) = 0.57, P-factor (p) = 0.0002) [11].

In a controlled clinical trial, 100 women were split into two equal groups (one being a low-fat diet group and the other a control diet group) and changes in BMI (27.93 ± 4.45 vs 26.05 ± 2.65; p = 0.01) and waist circumference (99.92 vs. 91.59 cm; p = 0.0001) were noted. This adds further evidence to the linkage between high-fat diets and obesity. Adipose tissue, through androgen aromatization, becomes a source of estrogen production. The same study also found that the low-fat diet group had a reduced estradiol (an endogenous estrogen) concentration (21.23 ± 14.32 vs 16.05 ± 10.25 ng/mL; p < 0.001) [23]. Saturated fat (based on many pre-clinical and epidemiological studies) has also been linked to increased levels of estrogen and insulin-like growth factor-1 (IGF-1) [14]. Estrogen appears to greatly influence the progression and mortality of hormone receptor-positive breast cancer [23,24]. Higher amounts of adipose tissue can also lead to the production of ROS as well as tumorigenesis via insulin and IGF-1 [11,14].

There is strong reason to believe that an unhealthy diet rich in fat might lead to obesity, and that has a myriad of implications for breast cancer, including worsening risk and affecting the quality of life and survival [1,28]. In the body, specific peptides called "adipokines" or "adipocytokines" are secreted by adipose cells, such as adiponectin and leptin. Adiponectin had an inverse correlation with obesity, whereas leptin had a direct correlation. A diet rich in saturated fat had an unfavorable adiponectin concentration and a higher leptin concentration, thereby intimating the association between this type of diet and obesity [4].

The impact of obesity is so significant that it has been shown to increase the risk of breast cancer-specific mortality by 18%, 14%, and 29% for every 5 kg/m2 BMI increase before, less than 12 months after, and more than 12 months after the diagnosis, respectively [14].

A study in Malaysia [25] involving 128 breast cancer survivors was conducted by measuring high molecular weight (HMW) adiponectin concentrations. Dietary patterns were analyzed, and it was found that for every single unit increase in an "energy-dense, high saturated fatty acid, low fiber" dietary pattern z-score, there was a reduction of 0.41 μg/mL in HMW adiponectin. This evidence supports the claim of dietary influence on adiponectin levels. Regarding leptin, elevated levels in obese individuals seem to pose a particular problem due to the overexpression of leptin receptors in breast cancer patients. Leptin affects apoptotic signals, inflammation, and estrogen sensitivity [4]. Adiponectin sensitizes tissues to insulin, so lower levels lead to hyperinsulinemia. This causes increased cell proliferation and inhibits apoptosis [23]. These combined factors have contributed to enhanced tumor growth and metastasis as well as increased mortality [25].

Blücher [10] explained that breast cancer cells have increased activation of lipid synthesis pathways and hyperactivity of the enzymes fatty acid synthase (FAS) and monoacylglycerol lipase (MGL). These allow for de novo lipogenesis (fat synthesis) and the release of intracellular fatty acids (FA). FAS and MGL, through lipogenesis and lipid mobilization, promote both tumor growth and aggressiveness. Breast cancer tissue, compared to healthy normal tissue, also showed an amplified integration of endogenous fatty acids. There is also an association between this membrane lipid configuration and the progression, mortality, and hormone receptor status of tumors, with a heavier lipid concentration in estrogen receptor (ER)-negative and grade three subtypes. These were the findings in terms of de novo fatty acid synthesis. The connection with dietary fat comes into play when we discuss patients who suffer in the long term and who often have cachexia, which is a general term to discuss the wasting of the body seen in many individuals with chronic diseases.

In patients with cachexia, the body is in a state of catabolism, and the body fat quantity is reduced through lipase enzymes (adipose-triglyceride lipase and hormone-sensitive lipase) that break down the triglycerides into FFA. The rising levels of FFA act as a foundation for cancer growth and tumorigenesis (through lipid signaling) [10]. Furthermore, a high-fat diet increases levels of chylomicrons in the intestine. These chylomicrons enter the circulation and cause the generation of free fatty acids, which are taken up by the liver. These hepatic-free fatty acids may enter the mitochondria for β-oxidation or be esterified into triglycerides [11]. Therefore, the body fat reserves and not just lipogenesis are sources by which breast cancer cells could harvest FFA in the diet to use for their growth [10].

**Figure 2:**
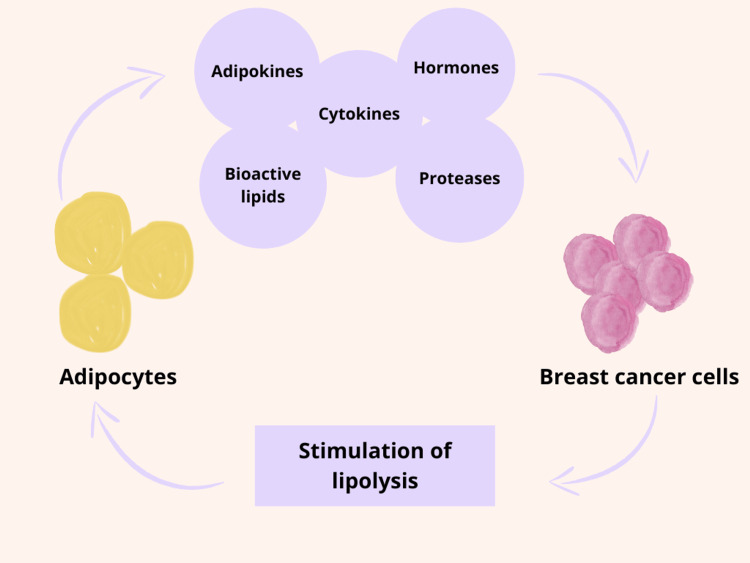
The cyclical interaction between adipocytes and breast cancer cells Figure inspired by Blücher and Stadler [10].

Figure 2 depicts the interaction between adipocytes and breast cancer cells. As certain compounds (adipokines, tumor necrosis factor-alpha, cytokines, hormones, and proteases) are released by adipocytes, breast cancer cells evolve into a more aggressive version. The cancer cells then stimulate lipolysis of the adipocytes, causing the release of inflammatory cytokines, proteases, and free fatty acids. This further progresses breast cancer. In obese individuals, there is oxidative stress, endoplasmic reticulum stress, increases in IGF-1 and leptin, as well as a decrease in adiponectin, which further promotes cancer [10,23].

As shown by a model created by Nieman and his colleagues [10], cancer cells can utilize fatty acids from fat cells as well. Another model by Balaban et al. [10] showed the proliferation of breast cancer cells with increased availability of fatty acids for mitochondrial beta-oxidation provided by adipocytes that mimicked those found in obese individuals (done by loading the co-cultured adipocytes with palmitate, oleate, and linoleate). This clearly shows the adaptive capability of cancer cells to their environments by activating pathways to support their growth (such as using exogenous lipids for cancer cell lipid synthesis and mitochondrial oxidation). Another study by Camarda et al. [10] showed triple-negative breast cancer cells with enhanced rates of fatty acid oxidation. Targeting these pathways slowed tumor progression by inhibiting energy metabolism.

Fat Quality

Now that we have explained the possible mechanisms by which dietary fat and breast cancer could be linked, let us delve into the finer aspects of this subject. It is important to remember that the harmful effects of dietary fat are not exclusive to all types of fat. It is critical to consider the quality, subtype, and quantity of fat when discussing potential interactions and potentially pathogenic mechanisms [1,10]. Let us first subdivide fat into separate categories and assess the involvement of these different varieties.

**Figure 3:**
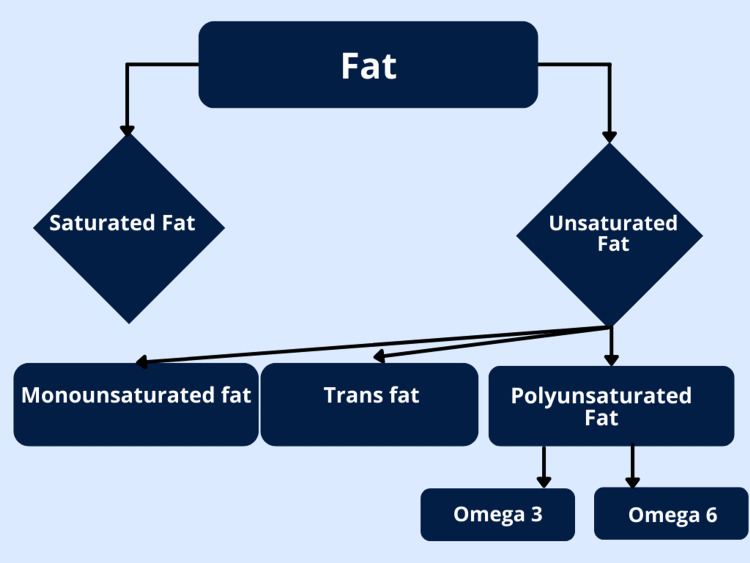
The classification of fats Figure inspired by Cenker [15].

By chemical structure, fats can generally be classified into saturated fats (SF), unsaturated fats (USF), and trans fats. USF is divided into monounsaturated (MUFA) and polyunsaturated (PUFA) fatty acids. PUFA are omega-3 fatty acids, which include alpha-linolenic acid (ALA), eicosapentaenoic acid (EPA), docosahexaenoic acid (DHA), and omega-6 fatty acids, which include linoleic acid (LA), arachidonic acid (ARA), gamma linoleic acid (GLA), and conjugated linoleic acid (CLA) [16]. The organization of these groups is depicted in Figure 3.

These various subtypes can have protective or harmful effects depending on which ones we consider and identify as part of our regular diet [1, 14].

Many studies have been conducted that show a positive and negative influence of fatty acids on cancer, whether it is harmful or protective. Elevated SFA, MUFA, and trans fats have been linked to an increased risk of cancer, whereas PUFA (such as omega-3 PUFA) have been shown to protect against it [1,10].

Comparing two diets that show varying levels of type and quantity of fat can help us resolve this matter. Western diets are known for having higher levels of saturated fat and trans fats than Mediterranean diets [12]. Mediterranean diets consisted of olive oil, vegetables, fruits, plant proteins, seafood, nuts, and low-fat dairy products, and had a fat composition that favored a higher amount of omega-3 and omega-6 PUFA [4,13]. The fact that Mediterranean diets are typically high in fat due to high olive oil consumption emphasizes the importance of quality over quantity in fat consumption [1]. A meta-analysis showed that breast cancer-specific risk was reduced among those following the Mediterranean diet (RR = 0.93 and 95% confidence interval (CI) = 0.87-0.99) [12].

Another case-control study, which had 10,000 cases of cancer and 17,000 controls, found a higher adherence to this diet decreased the risk of all types of cancer (an odds ratio (OR) of 0.76 for a two-point increment in their Mediterranean Diet Score) [15]. It also reduced the risk of non-breast cancer mortality in early-stage breast cancer survivors [28].

Many studies have shown that western diets, which have a lower omega-3 to omega-6 ratio, have a higher risk of several cancers [10]. They show an increasing trend of breast cancer incidence in developing countries with a high-calorie Western diet. A case-control study in Uruguay, for example, has shown an increased breast carcinoma risk (OR = 2.13, 95% CI = 1.09-4.15) in women consuming a western diet [15].

Many of these correlations between a western diet and breast cancer risk can also be partly due to red meat consumption. A pooled meta-analysis of case-control and cohort studies found that the RR of breast cancer for red meat consumption was 1.17 (95% CI = 1.06-1.29) [12].

Polyunsaturated Fats

While describing in detail the effects of PUFA, the dichotomy between the roles of omega-3 and omega-6 PUFA has to be highlighted. Omega-3 PUFA is said to be breast cancer (BC) protective and can reduce the risk by 5% for every 0.1 gram/day incremental increase in consumption of marine sources where they are found [14].

There are many theories regarding the mechanism of its protective effects. Due to the high peroxidizing capabilities of omega-3 PUFA, they are said to enhance chemosensitization. They rapidly integrate into cancer cell membranes (particularly those deficient in PUFA) and alter membrane integrity. They can even cause the generation of ROS and inhibition of anti-oxidation safeguards by cancer cells [14].

Subtypes of fats, such as EPA and DHA, are metabolized into resolvins and protectins and confer anti-inflammatory and immunoregulatory properties. In women with early-stage BC, these compounds could also protect healthy cells from chemotherapeutic agents [13]. A phase II trial involving 25 metastatic BC patients undergoing chemotherapy with anthracycline had shown improved survival among those whose plasma phospholipids were loaded with DHA (1.8 g/day), in addition to lower toxic effects of the chemotherapy itself [14].

Omega-3 PUFA could also bind to receptors within the nucleus of tumor cells, causing target gene modulation involving metabolism and cell death [14]. For example, EZH2 (enhancer of zest homologue 2), which is overexpressed in malignant breast tumors, is downregulated by omega-3 PUFA, resulting in reduced growth, proliferation, and metastasis [12].

In a cohort of women with early-stage BC, high eicosapentaenoic acid and docosahexaenoic acid intakes (>73 mg/day) from foods (marine sources) for 7.3 years reduced BC events by ∼25% and modulated BC risk biomarkers-both in premenopausal (Fabian et al., 2015) and postmenopausal (Hilakivi-Clarke et al., 2002) women-suggesting their potential contribution toward BC prevention [13]. Similarly, another meta-analysis of prospective cohort studies showed a 14% reduction in BC risk [12]. Eicosapentaenoic acid can be used to inhibit tumorigenesis via cyclooxygenase and lipoxygenase pathways, which lead to the production of leukotrienes, thromboxanes, and prostaglandins [10,14].

A phase III trial involving 65 BC patients with metastasis and focusing on the role of omega-3 PUFA and chemotherapeutic uses supported the discovery that DHA improves chemosensitivity. Future research into the chemoprotective effects of omega-3 PUFA and their therapeutic value in BC [14] may yield promising results. Systematic reviews and meta-analyses of the omega-3 to omega-6 PUFA ratio have demonstrated an inverse correlation with BC. This could be attributed to pro-inflammatory factors, oxidative stress, DNA damage, and enhanced insulin sensitivity factors, as well as an alteration in adiponectin levels [13]. 

On the other side of the spectrum, omega-6 PUFA is said to have pro-inflammatory and pro-tumorigenic inclinations. Higher omega-6 PUFA consumption (seen in western diets) has been associated with an increased risk of multiple different cancers [10]. In particular, increased incidences of BC were found with higher levels of omega-6 PUFA, particularly long-chain PUFA such as arachidonic acid (AA) [13]. This could theoretically be associated with converting AA into eicosanoids that stimulate inflammation [10]. The enzyme cyclooxygenase-2 (which converts long-chain PUFA into eicosanoids) is inhibited by aspirin, and in high omega-6 PUFA Western diets, this has resulted in lower rates of BC mortality and all-cause mortality (if taken prior to diagnosis). The accumulation of AA (due to cyclooxygenase inhibition) within the cells has protected against BC, inhibiting growth and proliferation while stimulating apoptosis [13].

A recent meta-analysis of 19 prospective cohort studies including 1,013,273 participants and 195,515 deaths showed that a 5% increase in energy from PUFA was associated with a 4% (RR = 0.96, 95% CI = 0.94-0.99) reduction in mortality from cancer [6].

Monounsaturated Fats

Oleate, an omega-9 MUFA, has shown an increased expression of angiopoietin-like factor-4 (ANGPTL4) in different types of cancer cells, including BC cells. ANGPTL4 has been known to stimulate BC invasion into the lung [10].

The effect of MUFAs has also been varied when discussing the nature of BC in a particular patient. For example, Li et al. (2014) [30] showed that adenosine monophosphate (AMP) and activated protein kinase (AMPK), both regulators of energy homeostasis, had an upregulation in activity when BC cells were treated with oleate. AMPK increases fatty acid oxidation and adenosine triphosphate (ATP) synthesis by disrupting energy homeostasis, causing increased proliferation, growth, and migration. Interestingly, this effect was seen in very aggressive metastatic cells, which could be due to, according to Hardy et al., enhanced proliferation via G-protein-coupled receptor-40. However, this effect was reversed in less metastatic cells. So the contrasting effects of oleate on the progression of highly metastatic BC cells versus less metastatic cells further complicate the issue of how fat affects BC [10].

Also, regarding MUFAs, there have been mixed results depending on their source in the diet. MUFAs derived from extra-virgin olive oil have been negatively correlated with BC risk. This finding is most likely due to the oxidative stability of MUFA, as well as the reduction of insulin resistance and the polyphenols (hydroxytyrosol and oleuropein aglycone) found in olive oil. These polyphenols showed reduced survivability in BC cells [13]. A population-based BC study called the Four-Corners Breast Cancer Study found that an increase in MUFA consumption reduced BC risk in postmenopausal women (P-value for trend 0.01) [15].

Interestingly, MUFAs that were derived from trans-fatty acids such as margarine were positively correlated with BC risk [13].

Saturated Fats

Saturated fat has been considered one of the highest-risk fats for BC incidence and has a plethora of evidence to support it. A recent report by the World Cancer Research Fund/American Institute for Cancer Research has brought forth consistent findings suggesting this idea [1].

Table 9 contains studies that show the data on this association in terms of hazard ratio (HR), odds ratio (OR), and risk ratio (RR) with a 95% confidence interval (CI).

**Table 9:** Studies showing the effects of saturated fat on breast cancer

Study type	Reference	Findings	HR/OR/RR ( 95% CI)
Meta-analysis	Brennan et al. (2017) [18]	An increased risk of breast cancer-specific death.	HR= 1.63 (CI= 1.19-2.24)
Meta-analysis	Smith-Warner et al. (2001) [19]	An increased risk of breast cancer incidence with the substitution of saturated fat with carbohydrates (weak association)	RR= 1.09 (CI= 1.00-1.19)
Meta-analysis	Boyd et al. (2003) [20]	An increased risk of breast cancer incidence.	RR= 1.19 (CI= 1.06-1.35)
Meta-analysis	Xia et al. (2015) [21]	An increased risk of breast cancer incidence.	OR=1.33 (CI= 1.02-1.73)
Meta-analysis	Lodi et al. (2022) [17]	An increased risk of breast cancer incidence.	RR=1.12 (CI= 1.03-1.21)
Cohort	Sieri et al. (2014) [26]	An increased risk of breast cancer incidence.	HR=1.28 (CI= 1.09-1.52)
Case-control	Sofi et al. (2018) [27]	An increased risk of breast cancer incidence.	OR=3.4 (CI= 1.4-8.1)

As supported by many of the studies given in the table, in addition to many others, there is strong reason to believe in the harmful correlation between saturated fat and BC incidence and mortality. But surprising evidence has been found that challenges this theory. In fact, palmitate, the most ubiquitous fatty acid in human circulation, actually inhibited tumor growth in certain in vitro studies [10].

This may also vary depending upon the age demographics and ethnicity of the population. Xia et al. [21] noted a higher risk of BC for Asians (OR = 1.17, 95% CI = 1.02-1.34), Caucasians (OR = 1.19, 95% CI = 1.00-1.41), and women belonging to the postmenopausal group (OR = 1.33, 95% CI = 1.02-1.73), in case-control studies assessing dietary saturated fat.

Trans Fats

Trans fats were also found to be incredibly harmful to BC patients, significantly affecting those consuming high levels of them.

The Nurses' Health Study II (NHSII) [1] showed a positive connection between trans fat levels and risk of BC comparing the high and low quartiles (OR = 2.33, CI 95% = 1.45-3.77, p = 0.007) in a case-control study involving 1588 women with similar ages and BMI. In line with this observation, another study by Hirko et al. [29] showed an increased risk as well (OR = 2.33, 95% CI = 1.45-3.77).

One meta-analysis [3] also showed that this effect seemed to only occur in postmenopausal women (pooled effect size: 1.37, 95% CI = 1.04-1.81, p = 0.02). Widespread data also shows that, although not specifically by BC, mortality rates were also worsened by trans fats in the diet. The previously mentioned meta-analysis [6] found that a 1% energy increment in trans fat in the diet was associated with a 6% increase in all-cause mortality (RR = 1.06, 95% CI = 1.01-1.10). This is also supported by a study from Beasley et al. (2011) [15], which shows how trans fat increases all-cause deaths (HR = 1.78, 95% CI = 1.35-2.32).

Overall Fat Intake

Let us analyze the Women's Health Initiative Dietary Modification Trial for information regarding the effect of total dietary fat intake without any discrimination based on its subtypes. It was a randomized, controlled clinical trial [22] (registered: NCT00000611) involving 48,835 women aged between 50 and 79 years with no previous diagnosis of BC and a dietary fat intake greater than 32% of their total energy intake. These women were randomly assigned to a comparison group or an intervention group based on their diet. The intervention group aimed to reduce the dietary fat intake to less than 20% of total energy intake with a recommendation for increased consumption of grains, fruits, and vegetables [22].

The results of this trial were consistent with many of the proposed findings in this review. At the 8.5-year checkpoint after the trial initiation, there was an 8% decrease in BC incidence in the intervention group, with insignificant findings for survival. However, after the median 16.1-year checkpoint was reached, the intervention group found a statistically significant reduction in all-cause mortality following BC diagnosis. By the end of the median 19.6-year checkpoint, BC incidence had reached 3,374 cases, and deaths after BC had still been reduced (1,011 deaths and HR = 0.85, 95% CI = 0.74-0.96). Deaths specifically attributed to BC had also been reduced (383 deaths and HR = 0.79, 95% CI = 0.64-0.97) [22]

Although this population mainly concerned postmenopausal women, it did show the necessity for a low-fat dietary pattern to improve the survivability of breast cancer.

Limitations

As explained, some of the limitations of this study arise due to the dynamic nature of the subtypes of fat and their effects on BC, making it challenging to analyze due to the contrasting effects of the different types of dietary fat. Additionally, data from the selected articles for review assumes that the dietary fat intake of their participants is uniform as a group and does not account for inaccurate reporting or food waste. This study also mentioned the linkage of dietary fat to obesity, which has increased BC risk and mortality rates and may indicate a confounding effect.

Another possible method by which the data can be skewed is that people consuming higher levels of dietary fat may also have a lower likelihood of keeping up with a healthier lifestyle and initiating any form of dietary control. Many diverse factors can also contribute to worsening the risk and prognosis for BC, whether it be physical exercise, other unhealthy habits (i.e., alcohol consumption and smoking), or lack of body fat control.

## Conclusions

Based upon all the evidence provided in this discussion, entailing all the hypotheses and collected data from studies, strongly supports the notion that fat impacts breast cancer negatively by increasing the risk of its incidence and reducing survival in those already diagnosed. However, as we have seen, the question is more complex due to the diverse nature of fats and how they interact with cancer cells. Some have been shown to be protective, whereas others have been shown to be harmful.

However, more studies are needed for an in-depth analysis of the role of nutrition in breast cancer and how it can be modulated to improve the prognosis of many cancer patients and avoid the development of cancer itself. As a result, it is possible that dietary control could be a critical intervention point for reducing worldwide incidences of breast cancer and mortality as a result.
